# The Relative Age Effect in Ice Hockey: Analysis of Its Presence, Its Fading and of a Reversal Effect among Junior and Professional Leagues

**DOI:** 10.5114/jhk/161573

**Published:** 2023-04-20

**Authors:** Jean Lemoyne, François Trudeau, Simon Grondin

**Affiliations:** 1 Department of Human Kinetics, Université du Québec à Trois-Rivières, Trois-Rivières, Canada.; 2 Groupe Interdisciplinaire de Recherche Appliquée en Santé (GIRAS), Université du Québec à Trois-Rivières, Trois-Rivières, Canada.; 3 Laboratoire de Recherche sur le Hockey, Université du Québec à Trois-Rivières, Trois-Rivières, Canada.; 4 School of Psychology, Université Laval, Quebec City, Canada.

**Keywords:** relative age effect, ice hockey, birth distribution, advanced analytics, performance, quantile regression

## Abstract

This study analyzes the relative age effect (RAE) among the world's best junior hockey leagues and in the NHL. Despite the prevalence of RAE in ice hockey, past research suggests its fading-reversal over time, which may occur at later stages of athletic development. The hypothesis of the RAE reversal was tested with two sources of raw data files from the 2021-2022 season: 15 of the best international junior and minor professional leagues (N = 7 399) and the NHL (N = 812). Birth quartile distributions were analyzed to verify the prevalence of RAE and quantile regression was used to test the reversal of RAE hypotheses. Advanced hockey metrics were aggregated from multiple data sources and used to compare early born with late born players using birth quartiles. Prevalence of the RAE was verified with crosstabs analyses and quantile regression was used to test the reversal effect. Results indicated that the RAE still prevailed in ice hockey, with higher magnitude in Canadian leagues. Regression analyses showed that late-born junior and minor pro players, despite getting less exposure in terms of games played, attained levels of offensive production similar to those of early born players. Late-born players able to emerge in the NHL performed similarly and sometimes displayed better performance (in some markers). Results suggest that stakeholders should find ways to pay special attention to late born players in talent identification processes and offer them opportunities to develop at the highest levels.

## Introduction

In sports, it is known that the moment of birth relative to the starting date of the eligibility period will have an impact on the chances of success (Musch and Grondin, 2001). This moment-of-birth effect, namely the “relative age effect” (Barnsley et al., 1985), reveals that, in the upper echelons of youth sport, the likelihood of participation is higher among those born at the beginning of the relevant selection period. Inversely, participation is more likely to be lower in those born at the end of the selection period. This effect holds even when the standard distribution of live births in a given population is taken into account (Grondin et al., 1984). The RAE seems to be more prevalent in high demanding sports where some aspects of physicality prevail and in which a physical advantage is valuable (Lames et al., 2009). Indeed, a recent study conducted among players from professional soccer leagues (> 5 000 players) in Europe showed that the RAE was observed across most leagues, in favour of early born players (Yagüe et al., 2018). Similar patterns were observed in Canadian (Nolan and Howell, 2010) and Swedish women’s elite hockey (Stenling and Holmström, 2014). Similar results were also found among European players (Bilgic and Devrilmez, 2021) where the RAE was found in Czech Republic’s minor hockey players (Bozděch and Zháněl, 2020), in Russian elite players (Bezuglov et al., 2020), and amongst players involved in the ice hockey world championship (Nykodým et al., 2020). More recently, Lemoyne et al. (2021) confirmed support for prevalence of the RAE in ice hockey, demonstrating that it was present among minor hockey players and across the country’s major junior leagues.

Despite the evidence of the RAE in ice hockey, there are reasons to question the long-term advantages of being born earlier in the competition year. In that respect, recent research suggests that the benefits of the RAE may be mostly short term, and late-born athletes could benefit from a reversal effect, in which it becomes possible for them to increase their potential to progress at a higher level (Gibbs et al., 2012). The mechanisms underlying the RAE seem to be preponderant at the selection level, where older and more mature players are more likely to be selected (Ashworth and Heyndels, 2007; Baker et al., 2010a; Işın and Melekoğlu, 2020; Sherar et al., 2007).

Such results allow for a deeper analysis of the potential reversal of the RAE that may occur at a higher level of play. Therefore, the aim of the present study was to identify whether there was a reversal of the RAE, and when it would occur, by taking a close look at the RAE across the world’s best junior leagues and at the NHL level.

### RAE and the Reversal Effect in Ice Hockey

Since it was studied in the work of Grondin (1982), the presence of the RAE in ice hockey has been demonstrated several times (Addona and Yates, 2010; Barnsley and Thompson, 1988; Barnsley et al., 1985). In Canadian minor hockey, the RAE has been observed at the midget (U17) elite levels and it suggests that the birth date is a standard which determines whether a player will compete at higher levels of competition. For example, Lavoie and colleagues (2015) showed that changing the cut-off date among minor hockey players systematically changed the birth distribution and that the return to the initial cut-off dates had the same impact on birth distribution (Lavoie et al., 2015). In general, the RAE suggests that early born athletes may benefit from being preferred to their younger counterparts by being perceived as more athletically gifted and being considered as higher performers (Musch and Grondin, 2001). This fact is not surprising, especially in ice hockey, considering its physical demands; it is reasonable to believe that more mature players may out-perform their competitors, will benefit from more exposure and will be offered more opportunities to be recognized as highly talented (Hancock et al., 2013; Sherar et al., 2007). Higher confidence (due to more playing time) and a more mature physical stature are critical factors that may account for the advantages of early born players in elite sport. From this perspective, being born “at the right time” seems to be a critical factor in determining whether a player will be selected on teams at higher levels (Baker and Logan, 2007), such as national selections or professional leagues (Deaner et al., 2013).

Despite the advantages of being born earlier in the competition year, some late-born players seem to be able to adapt and overcome their age disadvantage in the long-term (Ford and Williams, 2011). For example, the RAE tends to diminish when comparing a performance indicator such as the playing time of minor leaguers (Baker et al., 2010a). In this regard, many authors have suggested the presence of a “reversal” of the RAE (Gibbs et al., 2012; McCarthy et al., 2016) in which late-born players may get a return of the pendulum in their favour. Past research shows some empirical support for the RAE reversal hypothesis. Recently, Wrang and colleagues (2018) provide empirical support for a RAE reversal among Danish national handball players. More specific to ice hockey, Bryson et al. (2017) also provide some support for the RAE reversal at the NHL level. They demonstrated that, from the 1990–1991 to the 2007–2008 season, late born players tended to obtain more points and, globally, to have a better performance. In the same line, Fumarco et al. (2017) showed that among the 10% of the NHL’s best players, late-born players, even if less represented, tended to perform significantly better than early born players who were selected earlier in the entry draft. Such outcomes provide support for a reversal of the RAE in hockey, at least at the professional level. Two hypotheses were posited to explain the reversal effect in elite sport. The first hypothesis is related to a psychological mechanism and suggests that overcoming adversity and adapting to the challenges related with the fact of being younger during the athletic development process provide an opportunity to develop stronger motivation, resilience, and capacity to overcome obstacles. This was shown among a cohort of German elite soccer players (Ashworth and Heyndels, 2007) and seems probable among ice hockey players. The second hypothesis is related to biological factors. According to this hypothesis, the reversal effect occurs among those who have the genetic and athletic background that predispose young athletes for excellence (Ashworth and Heyndels, 2007; McCarthy et al., 2016). The biological-athletic hypothesis found empirical support in professional hockey showing that despite being drafted later, late-born players tended to be more productive and durable once established in the major league (Fumarco et al., 2017). In a more global perspective, Hancock et al. (2013) suggested that the RAE was established through a series of social interactions (involving coaches, scouts, parents and players themselves) that resulted in bias regarding player utilization, evaluation and future selection at higher levels. On a long-term perspective, these mechanisms may lead to reinforce late born players’ capabilities (or not) to adapt and overcome the potential disadvantages of the RAE. In fact, late born players who “survived” the early phases of elite sport might be better prepared in terms of competence, resilience and other such attributes, which suggest that they have the potential to outperform players who did not get this opportunity earlier in their career. Despite the plausibility of a reversal of the RAE in professional hockey, no study has yet given attention specifically to its moment of occurrence. Since most authors showed that the RAE still persisted at the major junior level, it is less clear to conclude that performance is affected in the same way as in the NHL. From this perspective, it seems that performance indicators used to measure the reversal effect (e.g., games played, points, draft rank) may not allow definitive conclusions to be drawn about the RAE and its reversal in ice hockey at both the junior and professional levels.

The purpose of this study was to verify the presence of the RAE and of its reversal in elite ice hockey. To provide a complete picture of the RAE in elite hockey, this study was conducted in two phases. In the first phase, the purpose was to verify the presence of the RAE across the world’s best junior leagues and European pro leagues in which players that were recently drafted or were still eligible for the NHL draft evolved. For this part of the study, we hypothesized that the RAE should be present in each of these leagues, according to what was shown in past research. For the second phase, the objective was to test for the presence of a reversal of the RAE in the same leagues and at the NHL level, on the basis of multiple performance indicators. Due to the exploratory nature of this investigation, we hypothesized that the RAE should be stronger in leagues where players were younger (junior). Inversely, and in continuity with past research on this topic, we posited that there would be a reversal of the RAE among professional players in the NHL. In this sense, late-born players should tend to display performances similar or higher than those of their older counterparts, as shown by Fumarco et al. (2017).

## Methods

### Research Design and Data Availability

This observational study was conducted using two samples. Sample 1 focused on the presence of the RAE among junior players and used raw data extracted from the website (pick224.com - data extracted from the website of Dave McPherson, 2019: https://pick224.com). Sample used in phase 1 consisted of 7 399 major junior and professional NHL eligible players who evolved in 15 different leagues, during the 2021–2022 season. These 15 leagues (see Table 1) distributed in 7 countries (Canada, Finland, Russia, Sweden, Czech Republic, Slovakia and the United States), were identified among the most important leagues worldwide, included the best junior players, and were cited as the main source for the NHL draft. Most leagues (73%: 11 out of the 15 leagues) were restricted to junior players, and the rest (27%; 4 leagues) were minor professional leagues in which junior and professional players evolved with professionals. Sample 2 was drawn from the 2021–2022 NHL regular season data (n = 812 players), extracted from the InStat^®^ database system. The InStat^®^ platform (https://instatsport.com/hockey) is a tool that provides advanced metrics calculated from game recordings. These data were then analyzed to determine if there was an association between physical skills and performance in a competitive setting. For both samples, we chose performance indicators that were available in both databases to test the reversal of the RAE at each level.

**Table 1:** Sample descriptive statistics.

League	Country	Level	N	Mean age (December 2021)
AHL	Canada / USA	Pro	803	26.06 ± 3.25
Allsvenskan	Sweden	Pro	317	26.65 ± 4.08
Czechia U20	Czech Republic	Junior	410	19.45 ± 0.90
Finland U20	Finland	Junior	529	20.23 ± 1.17
Liiga	Finland	Pro	364	26.54 ± 4.46
MHL	Russia	Junior	927	19.69 ± 1.06
NCAA	USA	College	1 256	23.30 ± 1.56
OHL	Canada	Junior	433	19.50 ± 1,24
QMJHL	Canada	Junior	403	19.70 ± 1.20
SHL	Sweden	Pro	327	27.96 ± 5.08
Slovakia U20	Slovakia	Junior	231	19.64 ± 1.16
Sweden U20	Sweden	Junior	487	19.61 ± 0.88
USDP	USA	n/a	37	18.25 ± 0.55
USHL	USA	Junior	392	19.73 ± 1.11
WHL	Canada	Junior	483	19.59 ± 1.24
NHL	International	Pro	812	27.48± 4.15

*AHL = American Hockey League; MHL = Molodiojnaìa Junior Hockey League; NCAA = National Collegiate Athletic Association; OHL = Ontario Hockey League; QJMHL = Quebec Junior Major Hockey League; SHL = Sweden Hockey League; USDP = United States Development Program; USHL = United States Hockey League; WHL = Western Hockey League; NHL = National Hockey League*.

### Variables and Measures

#### Birth Quartile and Anthropometric Measures

The birth quartile was calculated from raw data available in each database. For both studies, birth dates were coded into birth quartiles (Q1 to Q4) by referring to the usual categorization used in each league: 1) Q1: January–February–March, 2) Q2: April–May–June, 3) Q3: July–August–September, and 4) Q4: October–November–December. For anthropometric measures, body weight and height were used to verify if early born players still had a physical advantage over later born players (Q3 and Q4).

*On-Ice Performance Indicators*

Performance was assessed using metrics available in the data files. In Sample 1 (junior players), we assessed players’ performance using four performance metrics: 1) total number of games played, 2) total points (goals + assists), 3) 5-on-5 points, and 4) power play points. These indicators reflect a player’s utilization and their individual offensive contribution. In the second sample (NHL players), we used indicators that were collected from the InStat^®^ database. Since the InStat^®^ system provides more than 50 game metrics, we retained the following indicators: 1) total number of games played, 2) total points per 60 min of play, 3) plus-minus differential (per 60 min of play), 4) time on ice (e.g., playing time), 5) Corsi % (per 60), 6) Corsi against (per 60), and 7) expected goals (per 60).

**Plus/Minus Differential** was calculated by evaluating the difference between the number of goals when a player was on the ice (+) and the number of goals scored by the opponent when the player was on the ice (-). A positive score means that a player contributed positively to his team differential. Found (2016) showed that the plus-minus differential had good predictive validity for players’ success in ice hockey.

**Corsi Statistics**. The Corsi statistic was developed by ex-NHL goaltender, Jim Corsi, and is now recognized as a standard for evaluating players’ performance (Chan, 2012;**** Schwarzenbrunner, 2021**). Corsi** % results from the total shot attempts divided by the sum of “Corsi For” shot attempts and “Corsi Against” shot attempts. Corsi Against represents the amount of the opponents’ shot attempts when a player is on the ice. A Corsi% over 50% suggests that a player generates more shot attempts than the opponents, which suggests that this player has good contribution to his team puck possession.

**Expected Goals** (xG) is a statistic that evaluates a shot attempt or scoring chance with the likelihood of a goal. This metric is derived from an algorithm that considers multiple aspects such as shot location, the type of play that led to the shot and the goaltender’s save efficiency in the situation. Expected Goals was shown to be a valid indicator for players’ evaluation in professional hockey (McDonald, 2012).

### Statistical Analyses

All analyses were conducted with SPSS 28 software. In Phase 1 of the study, we tested for the presence of the RAE by conducting crosstabs analyses and calculating chi-square (χ^2^) scores for statistical inferences for both samples. Cohens’ W was also calculated to estimate effect size in the sample that would show a significant RAE. The magnitude of effect sizes was interpreted as small (*W* < 0.3), moderate (*W* = 0.5), and large (*W*= 0.8). We also compared players’ age and anthropometric data across birth quartiles by conducting one-way ANOVAs, with the Bonferroni correction for post hoc analyses. We assumed that the RAE would occur in cases where the proportion of early born players (Quartile 1) would be significantly higher than that of late-born players and would show no significant differences on anthropometric measures. More specifically, with NHL players we calculated the number of years it took players to attain the NHL, by subtracting the year they were drafted by the year they entered the league (Draft year - Entry in NHL), and dichotomized: 1) 0–3 years, 2) 4 years or more.

To analyse the reversal of the RAE, we used quantile regression. Compared to OLS regression, quantile regression is not limited by normality assumptions (Hao, 2007) and is optimal when comparing outcome variables that are strongly skewed and/or asymmetric. Quantile regression is also used to compare sub-samples and prevent selection bias that may be caused by arbitrary smaller samples (Lê Cook and Manning, 2013). Regression analyses were conducted on the 25^th^, 50^th^, 75^th^, and 90^th^ percentiles of each distribution. This technique was performed in the past to verify differences in professional soccer players’ salaries (Ashworth and Heyndels, 2007) and among the NHL players’ number of games played and total points (Fumarco et al., 2017). The RAE would be maintained if early born players are still higher-performing than the late-born players (significant regression coefficients at 75^th^ and 90^th^ percentile). Stabilization of the RAE would occur if no significant differences in performance is observed (no significant regression coefficient at the 75^th^ and 90^th^ percentile), and the reversal effect would occur if the performance of late-born players is significantly better than that of early born players at the highest performing quartiles (75^th^ and 90^th^).

## Results

### Age and Anthropometric Characteristics

Table 2 shows age characteristics and the morphological profile of the junior and NHL players. In Sample 1 (e.g., junior and minor pro leagues), no significant differences were observed when birth quartiles were considered (*F_(3)_* = 2.185, *p* = 0.088). Five leagues displayed significant age differences: WHL, QJMHL, Sweden U20, Finland U20 and Czechia U20. In each of these leagues, players who were born in Q4 tended to be younger, despite small effect sizes (all ε^2^ < 0.024). At the NHL level, Q4 players tended to be slightly older than Q1 players, but with a small, negligible effect size (*F* = 2.42, *p* = 0.065, ε^2^ = 0.009). Results from the group comparisons regarding players’ anthropometric characteristics revealed no significant differences for height (*F*_junior_ = 2. 14; *p* = 0.09; *F*_NHL_ = 1. 07; *p* = 0.36), and weight (*F*_NHL_ = 1.60; *p* = 0.583). This suggests that players from Q4 were not physically different from Q1, Q2, and Q3 players, at both the junior and NHL levels.

**Table 2:** Anthropometrical characteristics (mean ± standard deviation).

Birth Quartile	Age (years)		Height (m)		Weight (kg)	
	JR+ Minor Pr	NHL	JR	NHL	JR	NHL
Q1	21.95 ± 3.65	27.23 ± 4.13	1.83 ± 0.06	1.86 ± 0.06	n/a	90.81 ± 6.52
Q2	21.98 ± 3.65	27.06 ± 3.97	1.82 ± 0.06	1.86 ± 0.06	n/a	89.86 ± 7.32
Q3	21.91 ± 3.68	27.86 ± 4.33	1.82 ± 0.06	1.85 ± 0.05	n/a	89.38 ± 6.29
Q4	22.25 ± 4.19	28.00 ± 4.15	1.83 ± 0.06	1.85 ± 0.06	n/a	90.17 ± 7.79
Total	21.99 ± 3.75	27.48 ± 4.15	1.83 ± 0.06	1.86 ± 0.06	n/a	90.11 ± 6.97

*NHL: Q4 > Q1

### Prevalence of the RAE

The RAE was observed in all junior and minor pro samples (χ^2^_(df)_ = 130.04_(42)_; *p* < 0.001), with 33% of players born in Q1 and 16% in Q4. The RAE was also observed in the NHL sample (χ^2^_(3)_ = 22.24; *p* < 0.001). Further analyses revealed no RAE regarding the time players took to attain the NHL level (χ^2^_(3)_ = 6.4; *p* = 0.096). We took a closer look at the junior leagues by conducting crosstabs within each league separately. When we compared the distribution according to countries, there was a significant RAE for all countries, with higher proportions of Q1 players (χ^2^_Canada_ = 270.36; χ^2^_Finland_ = 39.35; χ^2^_Russia_ = 58.26; χ^2^_Sweden_ = 76.21; χ^2^_USA_ = 159.06; χ^2^_CZE_ = 12.05; χ^2^_SVK_ = 3.095, all at *p* < 0.001). Observed effect sizes were higher among Canadian leagues (*W* = 0.45), moderate in Finland (*W* = 0.21), Russia (*W* = 0.26), Sweden (*W* = 0.26), and in the United States leagues (*W* = 0.25). Effect sizes were smaller in Czech (W = 0.17) and Slovak (W = 0.12) leagues. The effect size was also small for the NHL sample (W = 0.16), suggesting that the RAE tended to fade at the professional level.

### Reversal of the RAE in the Junior Leagues

Results from the junior leagues regression analyses are presented in Table 4. The “games played” indicator reveals a significant RAE: Q1 players benefitted from more game time exposure, by playing more games than Q4 players (at 75^th^ and 90^th^ percentiles: *t*75 = 3.41; t90 = 2.96 (both at *p* < 0.001). For the other performance indicators, no RAE was significant, except for powerplay points with Q3 players collecting less points than Q4 players at the 75^th^ percentile: (*t* = −2.63, *p* = 0.008).

### Reversal of the RAE in the NHL

Results from the regression analyses conducted among the NHL players reveal that late born players who were able to emerge in the NHL tended to attain the same level of play that was observed among early born players (Table 4). Results from quantile regression at the elite standard (90^th^ percentile) showed that, compared to Q4 players, Q1 players displayed weaker performances on multiple performance indicators: Pts per 60 (*t* = −2.70, *p* = 0.007), and Time on Ice per game (*t* = −1.75, *p* = 0.07). As for other performance indicators, no significant difference was observed for games played (GP), plus-minus differential (+− diff), Corsi statistics (Corsi%, Corsi against) and expected goals (xG), which suggests that late born players displayed performances equal to, or better than those of Q1 and Q2 players.

**Table 3:** Prevalence of the RAE in junior and minor pro and the NHL.

Birth Quartile	JR N (%)	Residuals (observed-expected)	NHL N (%)	Residuals (observed-expected)
Q1	2 441 (33%)	591.5	248 (31%)	45.0
Q2	2 172 (29%)	322.5	221 (27%)	18.0
Q3	1 622 (22%)	–227.5	181 (22%)	–22.0
Q4	1 163 (16%)	–686.5	162 (20%)	–41.0
	χ^2^_(3)_ = 528.21; *p* < 0.001 Cohen’s W = 0.27		χ^2^_(3)_ = 22.24; *p* < 0.001 Cohen’s W = 0.16	

**Table 4:** Sample 1: Junior players’ performance based on birth quartile (results from quantile regression analyses).

Performance indicators	Birth Quartile (with 95% Confidence Intervals)				
	Constant	Q1	Q2	Q3	Q4
GP75	52 (50.58–53.42)	55 (1.28–4.72) *	53 (–0.75–2.76)	52 (–1.86–1.86)	52
GP90	61 (59.91–62.09)	63 (0.67–3.33) *	62 (–0.35–2.35)	62 (–0.43–2.43)	61
Pts75	28 (26.40–29.60)	27 (–2.94–0.94)	25 (–4.94– –1.02)	27 (–3.09–1.09)**	28
Pts90	40 (37.54–42.46)	42 (–0.98–4.98)	40 (-3.04–3.04)	41 (–2.22–4.22)	40
5v5-Pts75	19 (17.94–20.06)	19 (–1.29–1.29)	19 (–2.32–0.32)	19 (–1.39–0.39)	19
5v5-Pts90	28 (26.09–29.91)	29 (–1.32–3.32)	27 (–3.37–1.37)	28 (–2.50–2.50)	28
PP-Pts75	8 (7.29–8.71)	8 (–1.86–0.86)	7 (–1.88– –0.12) ***	8 (–0.93–0.93)	8
PP-Pts90	13 (11.91–14.09)	14 (–0.33–2.33)	13 (–1.35–1.35)	14 (–0.43–2.43)	13

* Q1 displayed better performance than other groups: p < 0.001. ** Q3 displayed lower performance than Q4: p < 0.001. *** Q3 displayed lower performance: p < 0.05. *GP: games played; Pts: Total points; 5v5-Pts: Points at even strength; PP-Pts: Points during power play situations*

**Table 5:** Sample 2 (NHL) performance based on the birth quartile (results from quantile regression).

Outcome Variables at	Birth Quartile (with 95% Confidence Intervals)				
	Constant	Q1	Q2	Q3	Q4
GP75	81 (77.94–84.06)	79 (–5.93–1.93)	79 (–6.03–2.03)	80 (–5.21–3.21)	81 (n/a)
GP90	89 (85.70–92.30)	88 (–5.24–3.24)	86 (–7.34–1.34)	88 (–5.54–3.54)	89 (n/a)
Pts-60–75	2.1 (1.87–2.33)	1.86 (–0.54–0.06)	2.00 (–0.41–0.21)	2.2 (–0.22–0.42)	2.1 (n/a)
Pts-60–90	2.9 (2.62–3.18)	2.4 (–0.86– –0.14) **	2.7 (–0.57–0.17)	3.0 (–0.29–0.49)	2.9 (n/a)
Plus-Minus 75 (per 60)	0.37 (0.19–0.55)	0.38 (–0.11–0.35)	0.36 (–0.29–0.18)	0.39 (–0.16–0.34)	0.37 (n/a)
Plus-Minus 90 (per 60)	0.89 (0.68–1.10)	0.77 (–0.36–0.15)	0.93 (–0.23–0.31)	0.91 (–0.27–0.31)	89 (n/a)
TOI/G75	19:00 (17:30–19:00)	18:03* (–2:04–0:06)	18:05* (–2:04–0:10)	18:03* (–2:08–0:12)	19:00 (n/a)
TOI/G90	21:04 (18:10–19:43)	21:07 (–2:12–0:48)	20:06 (–2.24–0:36)	20:05 (–2:36–0:30)	21:03 (n/a)
Corsi %-75	52.75 (51.67–53.82)	51.93 (–2.21–0.56)	52.53 (–1.63–1.19)	53.19 (–1.03–1.92)	52.75 (n/a)
Corsi %-90	55.66 (35.32–57.02)	55.29 (–2.10–1.35)	55.56 (–1.88–1.65)	55.45 (–2.07–1.63)	55.70 (n/a)
Corsi ag.-75	43 (41.78–44.22)	44 (–0.57–2.57)	44 (–0.61–2.61)	43 (–1.68–1.68)	43 (n/a)
Corsi ag.-90	40 (38.59–41.41)	41 (–0.82–2.82)	41 (–0.86–2.86)	41 (–0.94–2.94)	40 (n/a)
xG per 60–75	0.85 (0.89–1.06)	0.90 (–0.24– –0.02)	0.94 (–0.19–0.03)	0.98 (–0.15–0.07)	0.90 (n/a)
xG per 60–90	1.15 (1.04–1.25)	1.05 (–0.24– –0.03)	1.11 (–0.17–0.10)	1.13 (–0.16–0.12)	1.15 (n/a)

* p = 0.10; ** p < 0.01, GP: games played; Pts: Total points. Plus-Minus differential: difference between goals for and the goal against. A positive score means that the player has a positive contribution. Corsi %: (Corsi For / [Corsi For + Corsi Against]) * 100 Corsi Against (Corsi ag): the amount of the opponent’s shots towards a player’s team. xG per 60: Expected goals per 60 minutes of play: a statistic that is derived from shot location, play situation, opponent’s goaltenders save %. Expected goals represents a player’s potential contribution.

## Discussion

As shown in past research, the RAE in elite junior and professional ice hockey leagues is strong. This is not surprising considering that the RAE is particularly strong in sports that value physicality, such as soccer, rugby, handball and ice hockey (Baker et al., 2010b; de la Rubia et al., 2021). Most specifically in ice hockey, many authors have shown that the culture of this sport leaves more room for players who have an imposing physical stature. Sport popularity may also be a factor that could explain the persistence of the RAE. Only in Canada, more than 300 000 players take part in ice hockey, thus suggesting that early phases of talent detection practices might play in favor of taller, more mature, and physically dominant players. The other objective of this study was to look at the RAE magnitude across the world’s best junior (and minor pro) leagues and to determine if this effect could be observed in performance indicators. Another objective was to test whether the RAE faded at a later stage of development, even to the point of resulting in a reversal effect in different areas of performance (Gibbs et al., 2012; McCarthy et al., 2016).

The first part of our analyses confirmed that the RAE is still prevalent after 40 years of research focused on this topic. In the NHL, it seems that the RAE still prevails since the mid-fifties, and in very similar proportions (Wattie et al., 2007). In fact, the evolution of players’ morphology over the last few decades could explain why the RAE persists in the NHL. Even with the recent emergence of the “fast and skilled” player prototype, the return to the norms for “big and fast” players among recent Stanley Cup champions (Larkin, 2019) suggests that taller and bigger players are still prioritized. Our results suggest some interesting outcomes related to the prevalence of the RAE. In fact, the RAE is prevalent in the most elite leagues. Moreover, the RAE tends to prevail even in leagues that allow older players to evolve, namely Allsvenskan, Liiga, NCAA and SHL. Inversely, the RAE was stronger in leagues where players were selected at a younger age, such as the Canadian Hockey League and the United States Development Program (USDP), both networks representing the best North American NHL prospects. However, we must recognize that the prevalence of the RAE among the most competitive leagues was expected. In sports such as ice hockey, the talent identification processes start at an early age (e.g., 14–15 years old, even younger), which corresponds to a stage which gives a considerable advantage to more mature athletes. For example, Roczniok et al. (2013) showed that early born players were predominant in a cohort of elite adolescent hockey players. In fact, early born players displayed higher levels of physical attributes, which justified their selection. More recently, similar results were observed among cohorts of Canadian male and female players who took part in evaluation camps (Huard Pelletier and Lemoyne, 2022). In consequence of such results, the pool of available players for future drafts (e.g., junior, professional, national teams) came mostly from early born athletes. Findings from our study are congruent with those of Bryson and colleagues (2017) and suggest that players gaining benefits from the RAE have more chances to be drafted in elite junior leagues (and even in the NHL). The high amount of membership combined with popularity of the sport and high standards in recruitment are probably the main factors explaining why the effect sizes are higher in Canada. Even if a more specific investigation into the differences between each league’s talent selection is beyond the scope of this study, trying to understand how talent is detected at these levels of competition would be an interesting avenue for future research and could provide a better understanding of the importance given to other components of talent in ice hockey. In line with our results, this investigation appeal to give more attention to the ways to keep late born players into most hockey programs that put attention to talent identification processes.

The second part of our analyses aimed to take a closer look at the so-called reversal effect, which would start with a fading of the RAE in elite ice hockey. The introduction of modern hockey analytics provides an innovative way to look at the multiple facets of players’ performance. First, the data indicate that late-born players have anthropometric characteristics similar to those of their older counterparts. This finding is consistent with the results in past research involving NHL players, which showed no associations between birth quartile and body mass index (Fumarco et al., 2017). Despite such resemblances, early born players were still offered more exposure (more games played) and received (slightly) more opportunities in one-man advantage (power play points). This means that older players still have an edge when opportunities present themselves to get more exposure for talent detection. But, despite such advantage, late-born players were as effective as early born, even at even strength (5 vs. 5) offensive production. Such results at the major junior level could be interpreted as a starting point for the reversal of the RAE. Analyses conducted among the NHL sample are consistent with the hypothesis of reversal effects (Fumarco et al., 2017). In the present study, different performance indicators than those reported by Fumarco et al. (2017) were used. The new results support the hypothesis of a fading of the RAE in ice hockey. In this sense, in top players (percentiles 75^th^ and 90^th^), late-born players were at least equal, and on some occasions even better, than early born players on each variable under investigation. These data show that despite the early-stage disadvantage (e.g., draft status, getting less exposure at earlier stages), late-born players contribute more offensively, and show better overall performances. In summary, our results are in line with previous research (Bryson et al., 2017; Fumarco et al., 2017): late-born players, despite a bumpier path to excellence, tend to overcome obstacles and achieve levels of performance equal to or higher than those of players who were advantaged at the earlier stages of their hockey career. Seen from a different perspective, the reversal effect may also be interpreted as a demonstration that when late-born players are able to join the NHL, they are more likely than early born players to reach the NHL due to their hockey talent, not to some physical advantages or other advantages that wane over the years when facing players with the same age and playing experience.

## Limitations and Future Directions

This study shows that despite its presence in elite hockey, the RAE tends to fade over time, mostly at the NHL level, and there are significant indications of a reversal effect. Despite the contributions of this study, it has some limitations that will deserve attention at the moment of designing future research testing for the reversal effect hypothesis. It would be worthwhile to analyse more deeply the relationships between birth quartiles and psychological variables such as grit, resilience, and motivation of elite ice hockey players. In this regard, some work has been conducted among younger ice hockey populations (Huard Pelletier and Lemoyne, 2022), and it seems relevant to go further with players who evolve at higher levels of competition. Future research could also be inspired from qualitative designs, to verify how late born players tend to overcome obstacles in their pathway to professional hockey (Herbison et al., 2019). Note also that performance indicators were based on game statistics, which is a global approximation of performance evaluation. Future research should consider the usefulness of emerging technologies that are designed to assess more thoroughly multiple areas of performance. Such technologies allow the integration of more variables of performance outcomes. The use of a transversal design also has some limitations. Such analyses of the reversal effect should be conducted with prospective or longitudinal designs to go further in the understanding of the mechanisms involved in any reversal effect. In complement, qualitative methodologies, involving players, coaches, and scouts (e.g., semi-structured interviews, group discussions) should prove to be an interesting addition to shed light on the consequences and challenges associated with the RAE in ice hockey.

## Practical Perspectives

From this research some promising paths for action arise. In a practical sense, talent detection procedures should be revised or at least should innovate and think about ways to provide equal opportunities for players to attain the highest levels of excellence in their sport. Keeping an eye on late-born players one more year during crucial selection steps (e.g., junior draft year) could give them the opportunity to catch up and offer them more exposure. One avenue for giving equal opportunities to late born players would be to adopt some changes in junior league regulations. For example, junior leagues could allow late born players to be eligible for one more season, which would provide them the opportunity to continue their development. Another way to help the development of late born players would be to offer them to take part in showcases, camps or other talent identification events in which they could compete with younger early born players. Such approach would also be beneficial for the development of early born players who would have an opportunity to compete against older players. A closer observation into how and when the reversal takes place during the stages that lead to excellence could also help identify which aspects of performance should be assessed in the talent detection process. Researchers need to combine their efforts with federations and leagues to try to find scenarios that will be positive on players, on either the developmental or performance component.

## Author Contributions

Conceptualization: J.L. and S.G.; methodology: J.L.; software: J.L.; validation: J.L.; formal analysis: J.L.; investigation: J.L.; resources: J.L. and S.G.; data curation: J.L.; writing—original draft preparation: J.L.; writing—review & editing: F.T. and S.G.; visualization: F.T.; supervision: S.G. and F.T.; project administration: J.L. All authors have read and agreed to the published version of the manuscript.

## ORCID iD

Jean Lemoyne: 0000-0002-5766-2203
